# Aquatic Insects in Eastern Australia: A Window on Ecology and Evolution of Dispersal in Streams

**DOI:** 10.3390/insects2040447

**Published:** 2011-10-20

**Authors:** Jane M. Hughes, Joel A. Huey, Alison J. McLean, Olivier Baggiano

**Affiliations:** Australian Rivers Institute and Griffith School of Environment, Griffith University, Nathan QLD 4111, Australia; E-Mails: j.huey@griffith.edu.au (J.A.H.); alison.mclean@griffith.edu.au (A.J.M.); o.baggiano@griffith.edu.au (O.B.)

**Keywords:** phylogeography, Trichoptera, Ephemeroptera, stream hierarchy model, Australia, gene flow

## Abstract

Studies of connectivity of natural populations are often conducted at different timescales. Studies that focus on contemporary timescales ask questions about dispersal abilities and dispersal behavior of their study species. In contrast, studies conducted at historical timescales are usually more focused on evolutionary or biogeographic questions. In this paper we present a synthesis of connectivity studies that have addressed both these timescales in Australian Trichoptera and Ephemeroptera. We conclude that: (1) For both groups, the major mechanism of dispersal is by adult flight, with larval drift playing a very minor role and with unusual patterns of genetic structure at fine scales explained by the “patchy recruitment hypothesis”; (2) There is some evidence presented to suggest that at slightly larger spatial scales (∼100 km) caddisflies may be slightly more connected than mayflies; (3) Examinations of three species at historical timescales showed that, in southeast Queensland Australia, despite there being no significant glaciation during the Pleistocene, there are clear impacts of Pleistocene climate changes on their genetic structure; and (4) The use of mitochondrial DNA sequence data has uncovered a number of cryptic species complexes in both trichopterans and ephemeropterans. We conclude with a number of suggestions for further work.

## 1. Introduction

Studies of connectivity among natural populations of organisms are often conducted at two vastly different timescales, each addressing questions of connectivity for slightly different reasons. Studies that focus on contemporary timescales are usually attempting to answer questions about dispersal abilities and dispersal behavior of a species [1,2]. In other words, their primary goal is to answer ecological questions. In contrast, studies that focus on historical timescales are more interested in evolutionary or biogeographical questions. For example, their aim may be to use historical demographic analyses of species to explain their current geographical distributions [3].

In the last 30 years or so, molecular approaches have increasingly been used to address these questions at both contemporary and historical timescales. While allozyme electrophoresis was the marker of choice in much of the 1980s and 90s, mitochondrial DNA sequence data has become increasingly popular. Mitochondrial sequence information, because it is haploid, uniparentally inherited and largely non-recombining has proved especially useful for inferring genealogies and inferring historical processes.

Basically, early approaches used differences in frequencies of alleles among populations to infer the levels of connectivity among them. Populations that shared alleles in similar frequencies were assumed to be highly connected by gene flow, whereas populations with highly significantly different allele frequencies were thought to have poor connections, either due to low dispersal abilities of the species or because of geographic barriers separating populations [4-6]. With the development of methods for directly sequencing fragments of the DNA and advances in statistical approaches, particularly the development of coalescent theory, more detailed inferences could be made [7-9]. Furthermore, with the burst of mitochondrial sequencing, it has become clear that there are probably many more biological species out there than can be identified by morphology alone [10]. Also, using ideas of molecular clocks, it is now possible to infer the timing of certain historical events, such as population divergences and expansions [11-13].

In this review, we present a synthesis of work addressing both contemporary and historical processes in eastern Australian aquatic insects, specifically species in the orders Ephemeroptera and Trichoptera. The central questions are: how has genetic information contributed to our knowledge of contemporary and historical connectivity for these species and is there evidence of cryptic species in these two groups? We finish with a list of remaining questions and future directions.

## 2. Ecological Timescales

Although the use of molecular markers for studying patterns of connectivity among populations has been around for a long time, relatively little work had been done on aquatic insects until the mid-1990s. At that time, studies of drift in stream insects had led many ecologists to propose that stream drift by larvae was the major dispersal mechanism in aquatic insects and that flight by adults, which tended to live for only very short periods, was likely only to result in some movement back up towards the headwaters, counteracting downstream drift [14].

**Figure 1:**
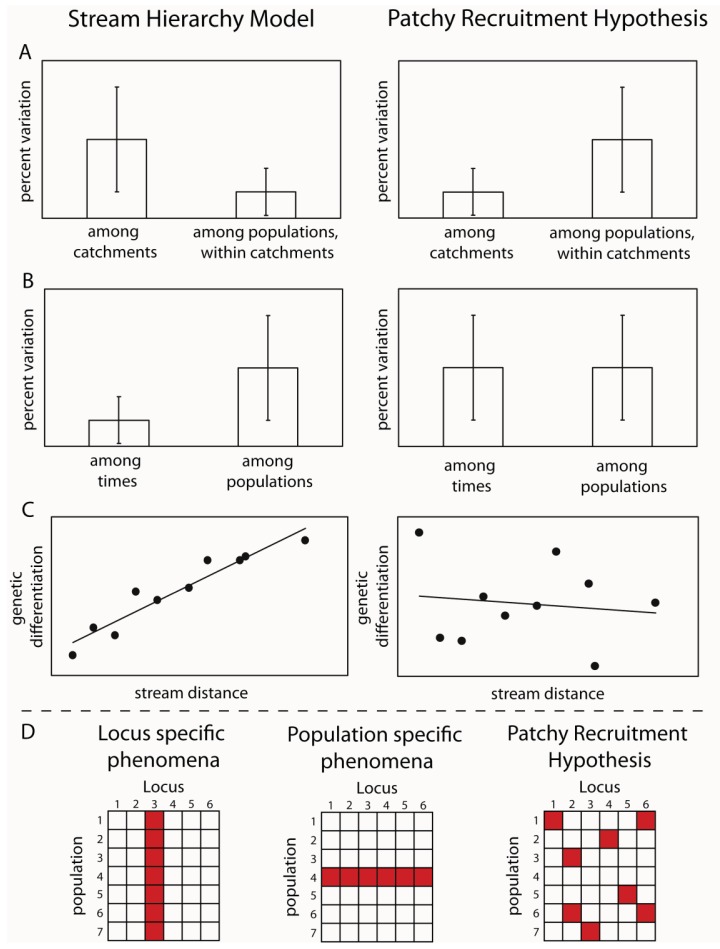
Predictions for population genetic variation under two contrasting demographic models; the Stream Hierarchy Model and the Patchy Recruitment Model (**A**–**C**); (**D**) Shows different interpretations of deviations from Hardy-Weinberg Equilibrium. Red squares indicate significant deviations.

By designing sampling programs that included sites from both within the same stream, from different streams in the same subcatchments and from across catchment boundaries, it was possible to test these hypotheses. The idea was that if larval drift was the major mechanism of dispersal, then genetic variation should reflect the stream hierarchy, in other words, should fit the stream hierarchy model [15]. Samples from the same streams should be more similar than those from different subcatchments and those from different catchments should be greater again (Figure 1). Alternatively, if adult flight is the major mechanism of dispersal, then genetic differences between sites would reflect the straight line distance between them. An initial study using a freshwater shrimp that is limited to the stream network demonstrated that the stream hierarchy model was adhered to; with the greatest genetic differentiation between two catchments, with differentiation decreasing between sub-catchments and further between streams within sub-catchments and pools within streams [16]. The reverse effect was shown for three aquatic insect species: *Bungona narilla*, *Tasiagma ciliata* and *Rheumatometra* sp., which each showed the most differentiation between pools within a stream and the least across catchment boundaries [1,17,18]. Clearly, genetic variation in these species did not fit the stream hierarchy model. While this was assumed to imply that adult flight was the major dispersal mechanism, the result of large significant differences between pools within streams was unexpected. Further observations of the genetic data indicated that the differences were not consistent among genetic marker loci (allozymes) and that there were numerous samples that did not fit predictions of Hardy-Weinberg Equilibrium (HWE). These deviations did not occur consistently at particular sites or particular loci. These findings led to the suggestion that the results could result from the fact that pools did not contain a representative sample of the genetic diversity because they represented the offspring of a limited number of females and that larvae did not move much within the stream. The small number of families in each pool would explain both the deviations from Hardy-Weinberg predictions and apparent differences between streams. This idea was labeled the “patchy recruitment hypothesis” (PRH) [1].

If patchy recruitment was the explanation for the patterns, then it was predicted that levels of differentiation and deviations from HWE should be random across loci and that any spatial pattern at one time would not be predictable from a previous time (Figure 1). In other words, we would expect as big differences between times at a site as between sites at a time. This was because a different small subset of families would be represented at different times. Our studies investigated these predictions and found strong support for them in both the caddisfly *Tasiagma ciliata* [17] and the mayfly *Bungona narilla* [19] in the Connondale region in southeast Queensland.

More recently, mitochondrial sequence data has been collected for *Tasimia palpata*, closely related to *Tasiagma ciliata* [20] and *Bungona narilla* [21] to further test predictions of the patchy recruitment hypothesis. Instead of allozyme frequencies, these studies used cytochrome oxidase I haplotype frequencies to determine whether there were larger differences between samples within catchments than between them.

The pattern observed for *Tasimia palpata* supported this prediction of the PRH, with greater differentiation among sites within streams than among sub-catchments or catchments. Because one of the implications of the PRH was that movement by larvae between pools was limited, the study of *Bungona narilla* also tested the hypothesis that movement of larvae within the streams would be more likely during wet periods than during dry periods, where there was very little hydrological flow connecting pools [21]. This was supported by mtDNA data, with significant differentiation among pools within a stream after a prolonged dry period, but not during a wet period.

The suggestion that only a few females contributed to the larvae in a particular pool was unexpected as many aquatic insects in the Northern Hemisphere are known to collect in mating swarms, which would make it likely that at any one time, many females would be ovipositing [22]. One explanation was that the situation in southeast Queensland was different because of the sub-tropical climate allowing adult emergence and oviposition over a more extended time period [23]. It was therefore proposed that if populations were sampled that emerged synchronously, as many Northern Hemisphere aquatic insects do; the effect may not be seen at all. *Tasimia palpata* occurs in both sub-tropical southeast Queensland and in temperate Victoria. While adults emerge throughout most of the year in southeast Queensland, in Victoria, adults of the species emerge in two synchronous “bursts” [23]. A comparison between temperate and sub-tropical populations of the same species showed significant genetic structure at the small scale in sub-tropical but not temperate populations and more than three times as many deviations from HWE in sub-tropical than temperate populations, at least partly supporting the idea that asynchronous adult emergence may be a factor leading to the genetic patchiness in Queensland populations. However, there was no evidence that fewer females contributed to a reach in Queensland than Victorian populations as there was no significant difference in the number of mitochondrial haplotypes per sample between the two regions. Schultheis *et al.* [23] suggested that the differences may be due to post-recruitment processes, such as non-random larval mortality and differences in the levels of larval drift between the two regions.

Clearly, more research is required to resolve the explanation for these patterns. With the development of hypervariable microsatellite markers, it may be possible to determine precise levels of relatedness between individual larvae within a reach. This has not yet been attempted for sub-tropical species, although Wilcock *et al.* [24] used microsatellites to assess relatedness between larvae of a net-spinning caddis in Britain. They found little evidence for patchy recruitment in their study, as near together larvae were not more closely related than expected from random. Clearly a similar study is needed in sub-tropical streams.

## 3. At What Scale are Populations of Caddisflies and Mayflies Connected?

Given that adult flight is the major mechanism of dispersal within the Conondale Range and that at that scale dispersal is widespread, we also wanted to know at what scale we might identify some genetic differentiation (*i.e.*, some constraints to dispersal). Here, we examine this question based on our studies of mayflies and caddisflies in eastern Australia.

Some studies of marine fishes have suggested that length of the dispersal phase (in their case, the planktonic larval stage) affects the level of genetic differentiation among populations [25,26]. Therefore, we have also examined the hypothesis that, in general mayflies are poorer dispersers than caddisflies, due to their extremely short adult life-stage [22]. Caddisflies are also stronger flyers than mayflies [27], which would also lead to a prediction of better dispersal abilities.

We used only those studies that had analyzed the mitochondrial gene cytochrome oxidase I [19,23,28-31]. We regressed overall *F*_ST_ values for each study against the maximum geographic distance between sites in the study. There was a significantly positive relationship (r = 0.56), with larger *F*_ST_ values at larger spatial scales, as would be expected. Based on that relationship, there should be an *F*_ST_ of at least 0.1 at spatial scales greater than 64 km, and an *F*_ST_ of 0.25 (less than one migrant between populations per generation) at spatial scales greater than 146 km. These distances are slightly larger, although the same order of magnitude as those calculated for a broader set of aquatic insects from studies from all over the world [32], which found a value for 10 km for an *F*_ST_ value of 0.1.

**Figure 2:**
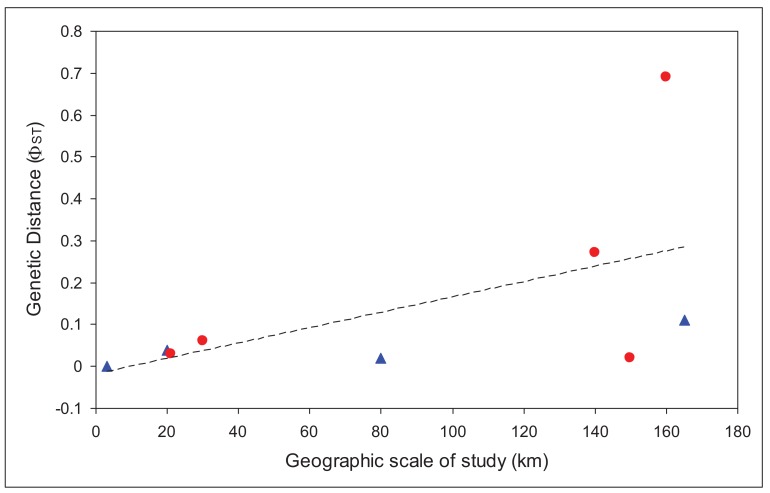
Scatter plot showing correlation between genetic distance and geographic scale of study for nine species of Ephemeroptera (red circles) and Trichoptera (blue triangles).

In order to account for the different scale of studies included in the analysis, we then compared the residuals for caddisflies with those for mayflies. Values above the line would indicate poorer dispersal abilities than the average, while those below the line would suggest better dispersal abilities. Figure 2 shows that all, except one, mayflies are above the line. This one point (*Atalophlebia* AV13 D) [29] is unusual in that it is the only truly lowland species in the analysis (for both mayflies and caddisflies). Nonetheless, these results strongly suggest that mayflies are poorer dispersers than caddisflies, at least in upland species. More studies, especially from other regions would enable a full statistical analysis of this trend and confirmation of the result.

## 4. Historical Timescales

Glaciation has been shown to have significant effects on genetic structure of terrestrial fauna in the Northern Hemisphere [33]. While glaciation was less common in the Southern Hemisphere [34], global climatic fluctuations at this time have been identified as a key driver of intraspecific diversification in Australia [35]. Many aquatic insect species living in upland rainforest and wet sclerophyll areas would have experienced cooler, dryer conditions, and their habitats were almost certainly reduced in size and displaced further upstream [36]. Four aquatic insects have been examined in this area at a broad scale of about 150 km [29-31]. These species vary slightly in habitat specificity, with two species, a caddis (*Tasiagma palpata*) and a mayfly (*Bungona narilla*) being strictly upland species, while the other two, both mayflies (*Atalophlebia* AV13 A and D), are found at lower elevations.

**Figure 3:**
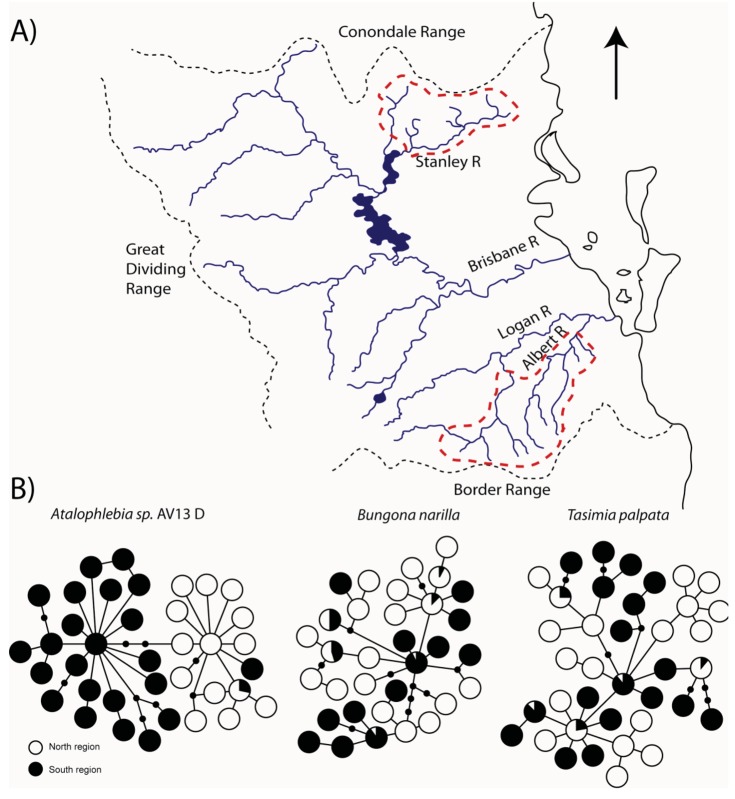

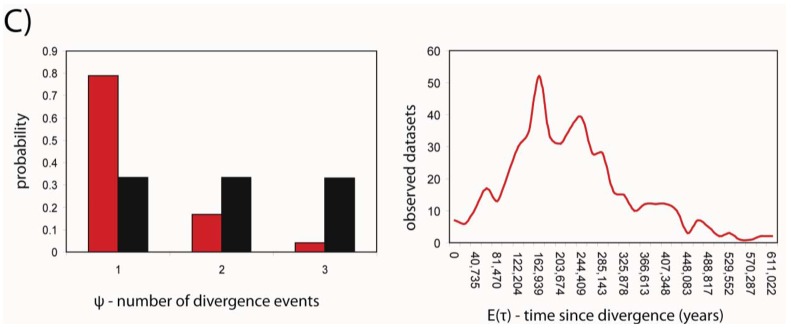
(**A**) Map of Conondale (north) and Lamington (south) regions; (**B**) Cytochrome Oxidase 1 haplotype networks for three species sampled in both north and south regions [29-31]; (**C**) We used a hierarchical Approximate Bayesian Computation method, analyzed with MTML-msbayes [38], to estimate the number of divergence events (Ψ) that can explain the shared pylogeographic break in these three co-distributed taxa. Within the msbayes pipeline, we simulated 1,000,000 datasets (parameterized by random draws from predefined ranges) and calculated 23 different summary statistics from each dataset to create a prior distribution. The same summary statistics were calculated for our observed datasets (based on the CO1 region of the mtDNA molecule) and compared to the simulated summary statistics to generate posterior distributions for the variance of τ (time since divergence), average τ and Ψ. The posterior distributions shown here for E(τ) and Ψ were estimated using the local regression method. For the histogram describing Ψ, the red bars show the posterior distribution while the black bars show the prior distribution. For a more detailed explanation of msbayes see [12,38,39].

The upland habitats (all above 200 m) where *T. palpata* and *B. narilla* occur are separated in southeast Queensland, by a lowland area of about 100 km, which may have presented a significant barrier to dispersal during glacial periods because it was dryer and even more inhospitable than it is currently. Both species occur in the Conondale, D'Aigular, Blackall Ranges north of Brisbane and in the Lamington and Great Dividing Range south and west of Brisbane (Figure 3). These upland areas have been labeled a “mesothermal island archipelago in a sea of subtropical lowlands” [37], and it was hypothesized that mitochondrial sequences would show evidence for historical isolation of these upland islands. Both studies used a fragment of the cytochrome oxidase I gene. As predicted there were highly significant differences in genetic composition between these two regions, suggesting that currently dispersal is very restricted (*T. palpata F_Ct_* = 0.10, p < 0.01, Φ_ct_ = 0.11, p < 0.01; *B. narilla F_Ct_* = 0.11, p < 0.001, Φ_ct_ = 0.17, p < 0.001). For both species there were two clades. However, each clade was not entirely restricted to the northern and southern regions. McLean *et al.* [30] hypothesized that for *B. narilla* this may be due to a recent range expansion. However, distinguishing between gene flow and incomplete lineage sorting with one gene is difficult.

For the other two species, *Atalophlebia* AV13 A and D, the results were rather different. Although the initial aim for this study was to examine lowland species, the two species were found at slightly different altitudes. *Atalophlebia* AV13 A was collected across a broader range of elevations, from almost sea level, to 200 m, whereas *Atalophlebia* AV13 D was only collected above 100 m. Interestingly, the patterns for *Atalophlebia* AV13 D were very similar to those seen for the two upland species. The *F*_ct_ between regions was 0.22 and the Φct was 0.67. There were two distinct clades, with one restricted to the south and the other almost totally restricted to the north. For *Atalophlebia* AV13 A, a very different pattern was observed. The *F*_ct_ was 0.02 and non-significant and the Φ_ct_ was 0.02 and only just significant. There was no evidence of past isolation in the network. This result was interpreted to mean that this species, with a largely lowland distribution and habitat preference was not constrained by the lowland separating northern and southern forested areas.

For the three species that were found to show evidence for historical isolation between the northern and southern regions (*T. palpata, B. narilla* and *Atalophlebia* AV13 D) two important questions follow. First, for all three species, did the northern and southern regions diverge at the same time? Given their shared distributions and similar habitat requirements, it is likely that the same climatic changes would have driven population divergence in all three species, producing comparable signatures in the population genetic data. Despite the networks showing subtly different patterns (e.g., *Atalophlebia* sp. AV13 D shows less sharing between two clades than the other species, possibly suggesting an older divergence time) it is necessary to analyze all three species simultaneously to account for coalescent stochasticity and differences in effective population sizes for each species [12]. The second question is; if all three species are the product of a single divergence event (or if there is evidence for multiple divergence events), when did it occur and what are the possible climatic drivers for this divergence.

Developments in statistical phylogeography have recently made the simultaneous analysis of shared phylogeographic breaks in co-distributed taxa possible. We used the msBayes pipeline [38,39] to estimate two important hyper-parameters for the three species; ψ and τ, the number of divergence events and the average time since population divergence, respectively. MsBayes suggested that a single divergence event was the most probable given the observed data, and that this divergence event occurred approximately 152 thousand years before the present (Figure 3). The 95% credibility intervals for the estimate of τ were 17,000 and 481,000 years before present.

The credibility intervals were wide; however the ability of msBayes to estimate these parameters accurately is reduced owing to the large influence of stochasticity in the coalescent at recent time scales [12]. Furthermore, the model used to simulate datasets was constrained to ignore migration. With only one marker (mtDNA), msBayes will perform poorly if estimating migration and divergence simultaneously [38]. The addition of multiple markers (e.g., nuclear sequence data) would enable more accurate estimation of τ.

The most notable result is that despite apparent differences in the networks, all three patterns are likely to have been produced from a single divergence event. Furthermore, it appears that the time of this event is relatively recent, occurring sometime in the late Pleistocene. Specific information about Pleistocene climates for southeast Queensland is lacking. However, it is clear that most of the Australian continent was not subjected to large scale glaciation. Nevertheless, this period was characterized by alternating periods of cool dry periods (glacials) when available habitats would have contracted and warm wet periods (inter-glacials) when habitats would have spread [35]. This “Pleistocene forcing” [35] has been identified as a key driver of intraspecific divergence throughout this period in Australia.

In the Northern Hemisphere, interpretation of phylogeographic data concentrates on the role of glacial refugia in determining current species distributions and genetic divergence within species [33,40]. While the hostility of a glaciated region will play an obvious role in determining distributions and shaping genetic variation within species, the alternating contraction and expansion of habitats during the Pleistocene has a much more complex impact on interpreting species distribution and genetic variation on non-glaciated continents. For example, signatures of population and range expansions, correlating with Pleistocene refugia, are often detected in Northern Hemisphere species [41]. For the species studied in Australia, population and range expansions have been detected, but dating of these events shows that some population expansions predate the divergence that has been detected among areas [31]. This suggests that the signatures of divergence, bottlenecks and population/range expansions during different climatic fluctuations are difficult to distinguish as the effects of these events are subtle. This is in contrast to heavily glaciated areas where the severity of bottlenecks and divergence in isolated refugia is likely to erase the genetic signatures of earlier events [40].

## 5. Cryptic Species

The identification of multiple biological species within a taxon currently classified as a single species is critical. Obviously, for conserving biodiversity, it is important to recognize that biodiversity before priorities can be made for its conservation [42]. Also, in order to understand the population biology of a species, it is critical that researchers are actually working with a single species. This is because, although individuals of cryptic species may be similar morphologically, they are likely to have very different biological attributes [43]. Furthermore, applied projects, such as the monitoring of species composition for assessing environmental health or bioassays for assessing toxicity of various compounds need to take into account the possibility of multiple species in their test organisms [44].

Recent work with mitochondrial DNA sequence data has provided evidence for the presence of cryptic species across many animal groups. Freshwater invertebrates are no exception. In our eastern Australian studies alone, we have found evidence for multiple cryptic species in two genera of atyid crustaceans, *Paratya australiensis* [45] and *Caridina indistincta* [46], freshwater crayfish, *Cherax dispar* [47] *and Tenuibranchiurus glypticus* [48] and freshwater mussels, *Velesunio ambiguous* [49]. In none of these studies was our aim to search for the presence of cryptic species. In each case, we were using mtDNA sequence information to infer past and present patterns of connectivity within species. However, high levels of divergence within mitochondrial gene trees is an indicator that cryptic species may be present in the sample [50].

**Figure 4:**
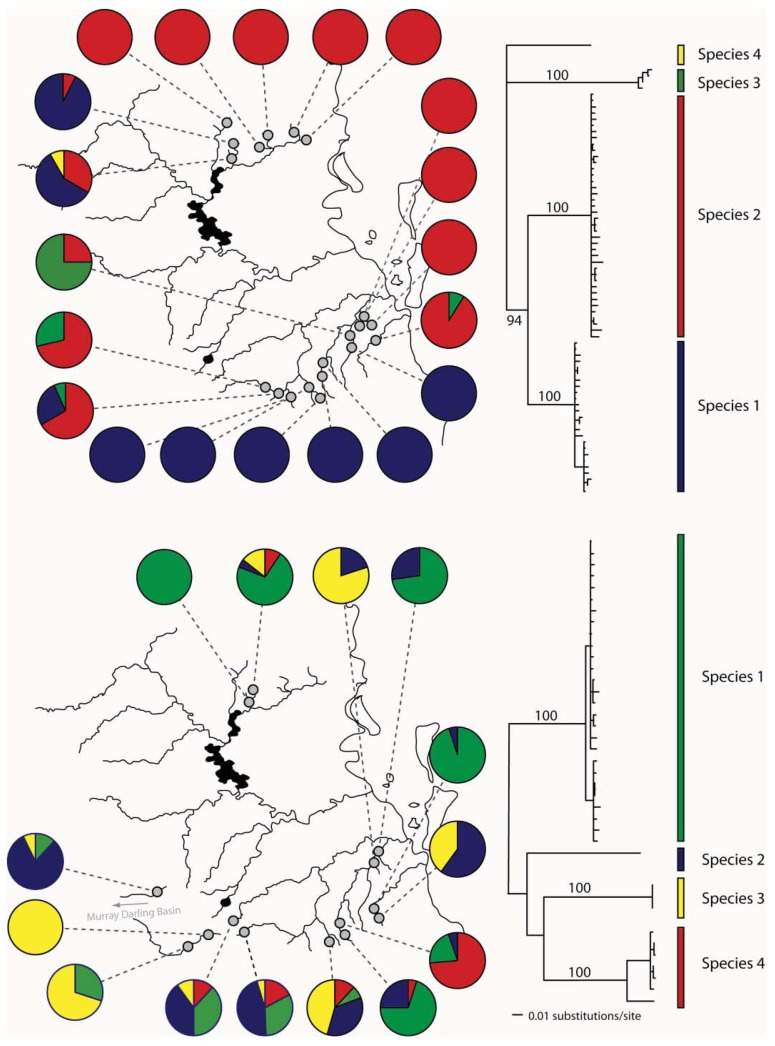
Phylogenies for two ephemeropteran taxa, *Altophlebia* spp. (top) and *Bungona narilla* (bottom), and the geographic distributions of cryptic species. The sampled region is identical to Figure 3.

In eastern Australian caddis and mayflies, we have also found evidence for cryptic species (Figure 4). In *Cheumatopsyche*, mtDNA COI indicated the possibility of multiple species in a sample from the Sydney area in New South Wales (NSW) [51]. We also identified *Atalophlebia* AV13A and D as being possible cryptic species from the mitochondrial DNA sequence data. Subsequent morphological analysis found some differences between species in both groups (John Dean personal communication). Further analysis of the two species of *Atalophlebia* showed that not only did they have rather different distributions, with one occurring from about sea-level to 200 m, whereas the other occurred only above 100 m, but also their genetic structure was very different, with one being essentially panmictic in the study area and the other highly structured between northern and southern regions. There were four species of *Cheumatopsyche* in the NSW study. One species, *Cheumatopsyche* AV1, was found in all 23 site sampled, while the other three species were only found in one, two or three sites [51]. Unfortunately, there is not always sufficient taxonomic expertise, so not all cases of cryptic species can be explored morphologically.

*Bungona narilla* is described as a single species in southeast Queensland [52]. Mitochondrial DNA sequence data suggested the presence of four distinct clades, which were about 20% divergent from one another. Subsequent allozyme analysis showed that each clade was distinct on the basis of four allozyme loci. A Bayesian analysis using the program STRUCTURE [53] identified four populations, with each correlating perfectly with the four mtDNA clades [32]. Unfortunately, the sample sizes so far have not allowed a detailed analysis of differences in distributions and genetic structure between the four species.

## 6. Conclusions

While substantial progress has been made in understanding the dispersal and connectivity among populations of a small number of ephemeropteran and trichopteran species in eastern Australia, many questions remain unanswered and further work is required.

1. Firm evidence for the patchy recruitment hypothesis requires the development of microsatellite markers to more accurately determine levels of relatedness between individuals in a pool.
2. A greater number of species needs to be added to the assessment of the differential dispersal abilities of Ephemeroptera and Trichoptera. Currently, only two families in each order have been examined.
3. In order to test the timing of divergence between populations more accurately, sequence data for a suite of nuclear genes needs to be included. The estimates presented in this study represent only the patterns from a single mitochondrial gene and any coalescent analyses lack power to discern a difference between different glacial periods. Furthermore, multiple genes allow for more complex phylogeographic histories to be tested, which is especially powerful using ABC methods.
4. The detection of many previously unknown cryptic species suggests many more are yet to be identified. As at least some of these cryptic taxa appear to be limited by temperature, it is imperative that they are recognized and their distributions understood, so that the potential impacts of future temperature increase can be predicted.
